# Correction to ‘Deliberation favours social efficiency by making people disregard their relative shares: evidence from USA and India’

**DOI:** 10.1098/rsos.170330

**Published:** 2017-05-10

**Authors:** Valerio Capraro, Brice Corgnet, Antonio M. Espín, Roberto Hernán-González

*R. Soc. open sci*. **4**, 160605. (Published 15 February 2017). (doi:10.1098/rsos.160605)

A reference was omitted from this paper and should be cited as reference [91] in §4.3, as follows:

‘Alternative approaches such as the Charness & Rabin [8] model would result in an identical classification of subjects, since the parameters of the basic Charness–Rabin model are linear transformations of the Fehr–Schmidt parameters [8,^91^].’

The updated reference list and data accessibility statement are shown below.

## Data accessibility

All data can be accessed at the Dryad Digital Repository http://dx.doi.org/10.5061/dryad.n581t [92].
