# Supplementary material for: Deliberation favours social efficiency by making people disregard their relative shares: evidence from USA and India
Source: R Soc Open Sci. 2017 Feb 15;4(2):160605. doi: 10.1098/rsos.160605 (PMC5367314; doi:10.1098/rsos.160605)
Supplement: The Supplementary Information includes details about the theoretical model, the statistical analysis, and the experimental instructions. [file rsos160605supp1.docx]

**Supplementary Information**

**The “model-based” definition of social preferences**

For the “model-based” definition of social preferences, we obtain meaningful ranges of values for the “envy” and “compassion” parameters of a generalized version of the Fehr and Schmidt (1999) inequality aversion model. Specifically, to characterize the motives underlying choices, we use a Fehr and Schmidt (1999) utility function which, for two players, denoted i and j, is characterized as follows:

$U_{i}\left( x_{i},x_{j} \right)=x_{i}-\alpha_{i}max \left\{ x_{j}-x_{i},0 \right\}-\beta_{i}max \left\{ x_{i}-x_{j},0 \right\}$,

where the parameters *α_i_* and *β_i_* refer to subject *i*’s *aversion* to disadvantageous (i.e. “envy”) and advantageous (i.e. “compassion”) inequality, respectively. We do not impose the parameter restrictions used in the original version of Fehr & Schmidt (1999)—that is, *α_i_* ≥ *β_i_* ≥ 0—so that we can generalize the categorization of participants beyond strict inequality aversion (egalitarianism). In particular, we classify individuals’ choices as follows:

(i) Socially *efficient*, if they maximize the total joint payoff (*α_i_* ≤ 0 and *β_i_* ≥ 0, with at least one inequality being strict, and *α_i_* > -0.5 or *β_i_* < 0.5);

(ii) *Egalitarian*, if they minimize payoff inequality (*α_i_* ≥ 0 and *β_i_* ≥ 0, with at least one inequality being strict);

(iii) *Spiteful*, if they maximize the decision maker’s relative standing by minimizing the other’s payoff (*α_i_* ≥ 0 and *β_i_* ≤ 0, with at least one inequality being strict);

(iv) *Self-interested*, if they maximize the decision maker’s own payoff (*α_i_* = 0 and *β_i_* = 0).

**Supplementary analysis**

***Main effects regressions***

Table S1. Social efficiency

|  | A. Trait level | |  |  | B. State level  All subjects | |  | C. State level  Inexperienced subjects | |
| --- | --- | --- | --- | --- | --- | --- | --- | --- | --- |
|  | Model-based | Choice-based |  |  | Model-based | Choice-based |  | Model-based | Choice-based |
| CRT score | 0.207*** | 0.244*** |  | Time delay | 0.477*** | 0.498*** |  | 0.938*** | 0.455 |
|  | (0.048) | (0.060) |  |  | (0.145) | (0.167) |  | (0.284) | (0.333) |
| India | -0.400* | -0.523** |  | India | -0.326** | -0.502*** |  | -0.336 | -0.722** |
|  | (0.218) | (0.245) |  |  | (0.152) | (0.179) |  | (0.274) | (0.335) |
| female | -0.096 | -0.162 |  | female | -0.323** | -0.477*** |  | -0.345 | -0.469 |
|  | (0.222) | (0.245) |  |  | (0.154) | (0.179) |  | (0.293) | (0.329) |
| age | -0.012 | -0.009 |  | age | -0.002 | 0.011 |  | -0.027 | -0.009 |
|  | (0.009) | (0.011) |  |  | (0.007) | (0.007) |  | (0.017) | (0.018) |
| Constant | -0.583 | -1.260*** |  | Constant | -0.067 | -1.135*** |  | 0.275 | -0.501 |
|  | (0.408) | (0.483) |  |  | (0.276) | (0.300) |  | (0.578) | (0.635) |
| χ^2^ | 22.565*** | 21.937*** |  | χ^2^ | 17.251*** | 22.334*** |  | 12.895** | 6.959 |
| pseudo-R^2^ | 0.090 | 0.128 |  | pseudo-R^2^ | 0.040 | 0.073 |  | 0.107 | 0.080 |
| ll | -111.782 | -83.309 |  | ll | -208.180 | -148.864 |  | -57.219 | -38.886 |
| N | 192 | 192 |  | N | 316 | 316 |  | 100 | 100 |

Notes: Probit estimates. Robust standard errors are presented in parentheses. Dependent variable: subject’s choices are classified as socially efficient. Main explanatory variables: CRT scores (between 0 and 7) in panel A, time delay (vs. time pressure) in panels B and C. * p<0.1, ** p<0.05, *** p<0.01

Table S2. Egalitarianism

|  | A. Trait level | |  |  | B. State level  All subjects | |  | C. State level  Inexperienced subjects | |
| --- | --- | --- | --- | --- | --- | --- | --- | --- | --- |
|  | Model-based | Choice-based |  |  | Model-based | Choice-based |  | Model-based | Choice-based |
| CRT score | -0.222*** | -0.127*** |  | Time delay | -0.394*** | -0.146 |  | -0.639** | -0.547** |
|  | (0.049) | (0.046) |  |  | (0.145) | (0.145) |  | (0.267) | (0.268) |
| India | 0.398* | -0.019 |  | India | 0.046 | -0.243 |  | -0.096 | -0.283 |
|  | (0.213) | (0.201) |  |  | (0.149) | (0.150) |  | (0.263) | (0.264) |
| female | 0.054 | 0.100 |  | female | 0.262* | 0.303** |  | 0.455 | 0.574** |
|  | (0.212) | (0.209) |  |  | (0.152) | (0.152) |  | (0.285) | (0.286) |
| age | 0.015 | 0.018* |  | age | -0.000 | 0.004 |  | 0.014 | 0.012 |
|  | (0.010) | (0.009) |  |  | (0.007) | (0.007) |  | (0.016) | (0.015) |
| Constant | 0.056 | -0.219 |  | Constant | -0.096 | -0.280 |  | -0.208 | -0.237 |
|  | (0.397) | (0.388) |  |  | (0.270) | (0.269) |  | (0.537) | (0.537) |
| χ^2^ | 23.352*** | 13.395*** |  | χ^2^ | 9.902** | 9.455* |  | 8.378* | 9.477* |
| pseudo-R^2^ | 0.101 | 0.051 |  | pseudo-R^2^ | 0.023 | 0.023 |  | 0.068 | 0.082 |
| ll | -119.648 | -126.330 |  | ll | -210.661 | -209.856 |  | -64.504 | -63.534 |
| N | 192 | 192 |  | N | 316 | 316 |  | 100 | 100 |

Notes: Probit estimates. Robust standard errors are presented in parentheses. Dependent variable: subject’s choices are classified as egalitarian. Main explanatory variables: CRT scores (between 0 and 7) in panel A, time delay (vs. time pressure) in panels B and C. * p<0.1, ** p<0.05, *** p<0.01

Table S3. Spitefulness

|  | A. Trait level | |  |  | B. State level  All subjects | |  | C. State level  Inexperienced subjects | |
| --- | --- | --- | --- | --- | --- | --- | --- | --- | --- |
|  | Model-based | Choice-based |  |  | Model-based | Choice-based |  | Model-based | Choice-based |
| CRT score | -0.161** | -0.182*** |  | Time delay | -0.135 | -0.489*** |  | -0.208 | -0.613** |
|  | (0.067) | (0.047) |  |  | (0.218) | (0.156) |  | (0.395) | (0.278) |
| India | 0.261 | 0.597*** |  | India | 0.829*** | 0.876*** |  | 1.270*** | 0.956*** |
|  | (0.267) | (0.207) |  |  | (0.233) | (0.158) |  | (0.454) | (0.290) |
| female | -0.210 | -0.327 |  | female | 0.523** | 0.229 |  | -0.001 | -0.136 |
|  | (0.263) | (0.215) |  |  | (0.232) | (0.163) |  | (0.456) | (0.284) |
| age | -0.012 | -0.004 |  | age | -0.009 | -0.008 |  | 0.041* | 0.023 |
|  | (0.016) | (0.011) |  |  | (0.012) | (0.008) |  | (0.024) | (0.018) |
| Constant | -0.474 | 0.173 |  | Constant | -1.754*** | -0.471 |  | -3.528*** | -1.208* |
|  | (0.592) | (0.445) |  |  | (0.464) | (0.296) |  | (0.856) | (0.640) |
| χ^2^ | 8.085* | 22.769*** |  | χ^2^ | 16.205*** | 38.841*** |  | 10.874** | 15.689*** |
| pseudo-R^2^ | 0.066 | 0.091 |  | pseudo-R^2^ | 0.104 | 0.108 |  | 0.136 | 0.119 |
| ll | -55.787 | -110.471 |  | ll | -76.126 | -175.935 |  | -26.131 | -59.626 |
| N | 192 | 192 |  | N | 316 | 316 |  | 100 | 100 |

Notes: Probit estimates. Robust standard errors are presented in parentheses. Dependent variable: subject’s choices are classified as spiteful. Main explanatory variables: CRT scores (between 0 and 7) in panel A, time delay (vs. time pressure) in panels B and C. * p<0.1, ** p<0.05, *** p<0.01

Table S4. Self-interest

|  | A. Trait level |  |  | B. State level  All subjects |  | C. State level  Inexperienced subjects |
| --- | --- | --- | --- | --- | --- | --- |
|  | Model- &  Choice-based |  |  | Model- &  Choice-based |  | Model- &  Choice-based |
| CRT score | 0.081* |  | Time delay | 0.031 |  | 0.504* |
|  | (0.046) |  |  | (0.144) |  | (0.269) |
| India | -0.123 |  | India | 0.138 |  | 0.268 |
|  | (0.208) |  |  | (0.149) |  | (0.265) |
| female | -0.153 |  | female | -0.269* |  | -0.597** |
|  | (0.215) |  |  | (0.154) |  | (0.290) |
| age | -0.021** |  | age | -0.010 |  | -0.006 |
|  | (0.009) |  |  | (0.007) |  | (0.016) |
| Constant | 0.072 |  | Constant | 0.146 |  | -0.266 |
|  | (0.403) |  |  | (0.275) |  | (0.553) |
| χ^2^ | 9.635** |  | χ^2^ | 7.384 |  | 8.130* |
| pseudo-R^2^ | 0.035 |  | pseudo-R^2^ | 0.018 |  | 0.074 |
| ll | -117.978 |  | ll | -211.251 |  | -62.292 |
| N | 192 |  | N | 316 |  | 100 |

Notes: Probit estimates. Robust standard errors are presented in parentheses. Dependent variable: subject’s choices are classified as self-interested. Main explanatory variables: CRT scores (between 0 and 7) in panel A, time delay (vs. time pressure) in panels B and C. * p<0.1, ** p<0.05, *** p<0.01

***Interaction (X country) effects regressions***

Table S5. Social efficiency

|  | A. Trait level | |  |  | B. State level  All subjects | |  | C. State level  Inexperienced subjects | |
| --- | --- | --- | --- | --- | --- | --- | --- | --- | --- |
|  | Model-based | Choice-based |  |  | Model-based | Choice-based |  | Model-based | Choice-based |
| CRT score | 0.252*** | 0.285*** |  | Time delay | 0.505*** | 0.650*** |  | 1.005** | 1.012** |
|  | (0.066) | (0.075) |  |  | (0.190) | (0.207) |  | (0.430) | (0.453) |
| India | 0.018 | -0.045 |  | India | -0.292 | -0.254 |  | -0.275 | -0.122 |
|  | (0.411) | (0.539) |  |  | (0.212) | (0.260) |  | (0.391) | (0.456) |
| CRT x India | -0.107 | -0.108 |  | delay x India | -0.066 | -0.433 |  | -0.122 | -1.211* |
|  | (0.093) | (0.115) |  |  | (0.294) | (0.351) |  | (0.560) | (0.665) |
| female | -0.085 | -0.133 |  | female | -0.324** | -0.491*** |  | -0.344 | -0.495 |
|  | (0.223) | (0.244) |  |  | (0.154) | (0.183) |  | (0.293) | (0.360) |
| age | -0.013 | -0.009 |  | age | -0.002 | 0.011 |  | -0.028 | -0.013 |
|  | (0.010) | (0.011) |  |  | (0.007) | (0.008) |  | (0.017) | (0.018) |
| Constant | -0.718* | -1.412*** |  | Constant | -0.077 | -1.213*** |  | 0.261 | -0.641 |
|  | (0.431) | (0.511) |  |  | (0.281) | (0.309) |  | (0.578) | (0.680) |
| χ^2^ | 23.613*** | 23.635*** |  | χ^2^ | 17.299*** | 25.117*** |  | 12.920** | 11.217** |
| pseudo-R^2^ | 0.095 | 0.132 |  | pseudo-R^2^ | 0.040 | 0.078 |  | 0.108 | 0.120 |
| ll | -111.194 | -82.893 |  | ll | -208.154 | -148.105 |  | -57.195 | -37.187 |
| N | 192 | 192 |  | N | 316 | 316 |  | 100 | 100 |

Notes: Probit estimates. Robust standard errors are presented in parentheses. Dependent variable: subject’s choices are classified as socially efficient. Main explanatory variables: CRT scores (between 0 and 7) in panel A, time delay (vs. time pressure) in panels B and C. * p<0.1, ** p<0.05, *** p<0.01

Table S6. Egalitarianism

|  | A. Trait level | |  |  | B. State level  All subjects | |  | C. State level  Inexperienced subjects | |
| --- | --- | --- | --- | --- | --- | --- | --- | --- | --- |
|  | Model-based | Choice-based |  |  | Model-based | Choice-based |  | Model-based | Choice-based |
| CRT score | -0.297*** | -0.205*** |  | Time delay | -0.440** | -0.131 |  | -0.873** | -0.619 |
|  | (0.071) | (0.064) |  |  | (0.190) | (0.188) |  | (0.406) | (0.401) |
| India | -0.150 | -0.609* |  | India | -0.007 | -0.225 |  | -0.273 | -0.337 |
|  | (0.396) | (0.370) |  |  | (0.205) | (0.207) |  | (0.365) | (0.362) |
| CRT x India | 0.157 | 0.167* |  | delay x India | 0.109 | -0.037 |  | 0.403 | 0.126 |
|  | (0.096) | (0.089) |  |  | (0.292) | (0.293) |  | (0.530) | (0.530) |
| female | 0.039 | 0.079 |  | female | 0.265* | 0.301** |  | 0.458 | 0.574** |
|  | (0.213) | (0.207) |  |  | (0.152) | (0.152) |  | (0.287) | (0.286) |
| age | 0.016 | 0.020** |  | age | -0.000 | 0.004 |  | 0.016 | 0.013 |
|  | (0.010) | (0.010) |  |  | (0.007) | (0.007) |  | (0.016) | (0.016) |
| Constant | 0.244 | -0.017 |  | Constant | -0.079 | -0.285 |  | -0.175 | -0.226 |
|  | (0.416) | (0.409) |  |  | (0.274) | (0.274) |  | (0.544) | (0.540) |
| χ^2^ | 24.118*** | 15.384*** |  | χ^2^ | 10.048* | 9.448* |  | 8.819 | 9.465* |
| pseudo-R^2^ | 0.111 | 0.064 |  | pseudo-R^2^ | 0.024 | 0.023 |  | 0.072 | 0.083 |
| ll | -118.239 | -124.596 |  | ll | -210.591 | -209.849 |  | -64.216 | -63.506 |
| N | 192 | 192 |  | N | 316 | 316 |  | 100 | 100 |

Notes: Probit estimates. Robust standard errors are presented in parentheses. Dependent variable: subject’s choices are classified as egalitarian. Main explanatory variables: CRT scores (between 0 and 7) in panel A, time delay (vs. time pressure) in panels B and C. * p<0.1, ** p<0.05, *** p<0.01

Table S7. Spitefulness

|  | A. Trait level | |  |  | B. State level  All subjects | |  | C. State level  Inexperienced subjects | |
| --- | --- | --- | --- | --- | --- | --- | --- | --- | --- |
|  | Model-based | Choice-based |  |  | Model-based | Choice-based |  | Model-based^#^ | Choice-based |
| CRT score | -0.187** | -0.146** |  | Time delay | -0.314 | -0.489** |  | - | -0.312 |
|  | (0.090) | (0.062) |  |  | (0.372) | (0.221) |  |  | (0.404) |
| India | 0.134 | 0.843** |  | India | 0.715** | 0.876*** |  | - | 1.148*** |
|  | (0.457) | (0.371) |  |  | (0.314) | (0.213) |  |  | (0.379) |
| CRT x India | 0.045 | -0.073 |  | delay x India | 0.274 | -0.001 |  | - | -0.478 |
|  | (0.132) | (0.091) |  |  | (0.465) | (0.312) |  |  | (0.530) |
| female | -0.215 | -0.321 |  | female | 0.533** | 0.229 |  | - | -0.138 |
|  | (0.264) | (0.214) |  |  | (0.227) | (0.163) |  |  | (0.282) |
| age | -0.011 | -0.004 |  | age | -0.008 | -0.008 |  | - | 0.020 |
|  | (0.017) | (0.011) |  |  | (0.012) | (0.008) |  |  | (0.017) |
| Constant | -0.418 | 0.084 |  | Constant | -1.702*** | -0.471 |  | - | -1.210* |
|  | (0.598) | (0.456) |  |  | (0.476) | (0.304) |  |  | (0.636) |
| χ^2^ | 8.516 | 24.013*** |  | χ^2^ | 16.888*** | 38.865*** |  | - | 16.260*** |
| pseudo-R^2^ | 0.067 | 0.093 |  | pseudo-R^2^ | 0.106 | 0.108 |  | - | 0.124 |
| ll | -55.725 | -110.180 |  | ll | -75.958 | -175.935 |  | - | -59.273 |
| N | 192 | 192 |  | N | 316 | 316 |  | - | 100 |

Notes: Probit estimates. Robust standard errors are presented in parentheses. Dependent variable: subject’s choices are classified as spiteful. Main explanatory variables: CRT scores (between 0 and 7) in panel A, time delay (vs. time pressure) in panels B and C. ^#^A probit model cannot be estimated in this case because there is only one individual (from the time pressure condition) classified as spiteful in US. OLS regression reports p-value=0.60 for the interaction term. * p<0.1, ** p<0.05, *** p<0.01

Table S8. Self-interest

|  | A. Trait level |  |  | B. State level  All subjects |  | C. State level  Inexperienced subjects |
| --- | --- | --- | --- | --- | --- | --- |
|  | Model- &  Choice-based |  |  | Model- &  Choice-based |  | Model- &  Choice-based |
| CRT score | 0.122** |  | Time delay | -0.105 |  | 0.374 |
|  | (0.061) |  |  | (0.191) |  | (0.423) |
| India | 0.209 |  | India | -0.020 |  | 0.162 |
|  | (0.380) |  |  | (0.206) |  | (0.361) |
| CRT x India | -0.090 |  | delay x India | 0.319 |  | 0.228 |
|  | (0.088) |  |  | (0.291) |  | (0.539) |
| female | -0.144 |  | female | -0.265* |  | -0.601** |
|  | (0.214) |  |  | (0.154) |  | (0.287) |
| Age | -0.021** |  | age | -0.009 |  | -0.005 |
|  | (0.009) |  |  | (0.007) |  | (0.016) |
| Constant | -0.044 |  | Constant | 0.200 |  | -0.232 |
|  | (0.421) |  |  | (0.281) |  | (0.553) |
| χ^2^ | 10.603* |  | χ^2^ | 8.765 |  | 8.959 |
| pseudo-R^2^ | 0.039 |  | pseudo-R^2^ | 0.021 |  | 0.076 |
| Ll | -117.496 |  | ll | -210.648 |  | -62.201 |
| N | 192 |  | N | 316 |  | 100 |

Notes: Probit estimates. Robust standard errors are presented in parentheses. Dependent variable: subject’s choices are classified as self-interested. Main explanatory variables: CRT scores (between 0 and 7) in panel A, time delay (vs. time pressure) in panels B and C. * p<0.1, ** p<0.05, *** p<0.01

***Accounting for numeracy skills***

Table S9. Effect of CRT controlling for numeracy

| Dependent variable: | 1. Social efficiency | | 2. Egalitarianism | | 3. Spitefulness | | 4. Self-interest |
| --- | --- | --- | --- | --- | --- | --- | --- |
|  | Model-based | Choice-based | Model-based | Choice-based | Model-based | Choice-based | Model- & choice-based |
| CRT score | 0.150** | 0.158** | -0.206*** | -0.167*** | -0.084 | -0.129** | 0.140** |
|  | (0.059) | (0.071) | (0.061) | (0.058) | (0.087) | (0.062) | (0.059) |
| Numeracy | 0.118 | 0.184** | -0.033 | 0.079 | -0.175 | -0.113 | -0.117 |
|  | (0.075) | (0.082) | (0.074) | (0.073) | (0.116) | (0.081) | (0.077) |
| India | -0.280 | -0.346 | 0.365 | 0.061 | 0.093 | 0.490** | -0.246 |
|  | (0.228) | (0.258) | (0.224) | (0.212) | (0.284) | (0.223) | (0.219) |
| female | -0.053 | -0.082 | 0.044 | 0.123 | -0.255 | -0.363* | -0.188 |
|  | (0.220) | (0.239) | (0.213) | (0.210) | (0.273) | (0.217) | (0.217) |
| age | -0.009 | -0.004 | 0.014 | 0.020** | -0.015 | -0.007 | -0.024** |
|  | (0.010) | (0.011) | (0.010) | (0.010) | (0.017) | (0.011) | (0.009) |
| Constant | -0.952** | -1.852*** | 0.160 | -0.463 | 0.006 | 0.520 | 0.432 |
|  | (0.471) | (0.557) | (0.456) | (0.448) | (0.693) | (0.506) | (0.464) |
| χ^2^ | 23.967*** | 23.483*** | 23.632*** | 14.585** | 9.372* | 25.138*** | 12.203** |
| pseudo-R^2^ | 0.099 | 0.150 | 0.101 | 0.055 | 0.087 | 0.099 | 0.044 |
| ll | -110.680 | -81.228 | -119.557 | -125.799 | -54.514 | -109.482 | -116.859 |
| N | 192 | 192 | 192 | 192 | 192 | 192 | 192 |

Notes: Probit estimates. Robust standard errors are presented in parentheses. Main explanatory variables: CRT score (between 0 and 7) and Numeracy score (between 0 and 6). * p<0.1, ** p<0.05, *** p<0.01

***Including subjects who did not respect the time constraints***

Table S10. Social efficiency

|  | A. State level  All subjects | |  | B. State level  Inexperienced subjects | |
| --- | --- | --- | --- | --- | --- |
|  | Model-based | Choice-based |  | Model-based | Choice-based |
| Time delay | 0.453*** | 0.470*** |  | 0.716*** | 0.563* |
|  | (0.130) | (0.151) |  | (0.239) | (0.293) |
| India | -0.302** | -0.393** |  | -0.109 | -0.582** |
|  | (0.134) | (0.155) |  | (0.238) | (0.290) |
| female | -0.287** | -0.343** |  | -0.319 | -0.419 |
|  | (0.138) | (0.160) |  | (0.249) | (0.295) |
| age | 0.001 | 0.016** |  | -0.016 | -0.017 |
|  | (0.006) | (0.007) |  | (0.015) | (0.016) |
| Constant | -0.223 | -1.394*** |  | -0.153 | -0.382 |
|  | (0.244) | (0.275) |  | (0.511) | (0.561) |
| χ^2^ | 18.631*** | 22.078*** |  | 10.769** | 8.716* |
| pseudo-R^2^ | 0.034 | 0.059 |  | 0.071 | 0.078 |
| ll | -261.307 | -187.523 |  | -78.423 | -51.934 |
| N | 396 | 396 |  | 133 | 133 |

Notes: Probit estimates. Robust standard errors are presented in parentheses. Dependent variable: subject’s choices are classified as socially efficient. Main explanatory variable: time delay (vs. time pressure). * p<0.1, ** p<0.05, *** p<0.01

Table S11. Egalitarianism

|  | A. State level  All subjects | |  | B. State level  Inexperienced subjects | |
| --- | --- | --- | --- | --- | --- |
|  | Model-based | Choice-based |  | Model-based | Choice-based |
| Time delay | -0.433*** | -0.241* |  | -0.557** | -0.450** |
|  | (0.129) | (0.130) |  | (0.229) | (0.228) |
| India | 0.015 | -0.274** |  | -0.244 | -0.390* |
|  | (0.133) | (0.134) |  | (0.231) | (0.230) |
| female | 0.254* | 0.218 |  | 0.357 | 0.309 |
|  | (0.137) | (0.138) |  | (0.243) | (0.243) |
| age | -0.002 | 0.007 |  | 0.019 | 0.014 |
|  | (0.006) | (0.006) |  | (0.013) | (0.012) |
| Constant | 0.009 | -0.310 |  | -0.242 | -0.206 |
|  | (0.236) | (0.235) |  | (0.463) | (0.453) |
| χ^2^ | 14.085*** | 14.531*** |  | 11.780** | 10.722** |
| pseudo-R^2^ | 0.026 | 0.028 |  | 0.072 | 0.065 |
| ll | -263.346 | -260.055 |  | -85.351 | -85.894 |
| N | 396 | 396 |  | 133 | 133 |

Notes: Probit estimates. Robust standard errors are presented in parentheses. Dependent variable: subject’s choices are classified as egalitarian. Main explanatory variable: time delay (vs. time pressure). * p<0.1, ** p<0.05, *** p<0.01

Table S12. Spitefulness

|  | A. State level  All subjects | |  | B. State level  Inexperienced subjects | |
| --- | --- | --- | --- | --- | --- |
|  | Model-based | Choice-based |  | Model-based | Choice-based |
| Time delay | -0.029 | -0.440*** |  | -0.094 | -0.432* |
|  | (0.195) | (0.137) |  | (0.315) | (0.229) |
| India | 0.616*** | 0.759*** |  | 0.758** | 0.643*** |
|  | (0.206) | (0.140) |  | (0.373) | (0.239) |
| female | 0.353* | 0.212 |  | 0.066 | -0.050 |
|  | (0.206) | (0.146) |  | (0.358) | (0.244) |
| age | -0.023* | -0.015** |  | 0.006 | 0.004 |
|  | (0.012) | (0.007) |  | (0.018) | (0.014) |
| Constant | -1.144*** | -0.137 |  | -2.004*** | -0.501 |
|  | (0.420) | (0.254) |  | (0.602) | (0.490) |
| χ^2^ | 12.803** | 42.065*** |  | 4.326 | 10.338** |
| pseudo-R^2^ | 0.076 | 0.091 |  | 0.059 | 0.063 |
| ll | -100.416 | -228.363 |  | -37.927 | -83.832 |
| N | 396 | 396 |  | 133 | 133 |

Notes: Probit estimates. Robust standard errors are presented in parentheses. Dependent variable: subject’s choices are classified as spiteful. Main explanatory variable: time delay (vs. time pressure). * p<0.1, ** p<0.05, *** p<0.01

Table S13. Self-interest

|  | A. State level  All subjects |  | B. State level  Inexperienced subjects |
| --- | --- | --- | --- |
|  | Model- &  Choice-based |  | Model- &  Choice-based |
| Time delay | 0.035 |  | 0.269 |
|  | (0.129) |  | (0.227) |
| India | 0.173 |  | 0.339 |
|  | (0.132) |  | (0.231) |
| female | -0.201 |  | -0.370 |
|  | (0.138) |  | (0.243) |
| age | -0.010 |  | -0.001 |
|  | (0.006) |  | (0.013) |
| Constant | 0.116 |  | -0.436 |
|  | (0.241) |  | (0.469) |
| χ^2^ | 9.092* |  | 6.540 |
| pseudo-R^2^ | 0.017 |  | 0.040 |
| ll | -265.702 |  | -85.808 |
| N | 396 |  | 133 |

Notes: Probit estimates. Robust standard errors are presented in parentheses. Dependent variable: subject’s choices are classified as self-interested. Main explanatory variable: time delay (vs. time pressure). * p<0.1, ** p<0.05, *** p<0.01

**Full experimental instructions**

Here we report full experimental instructions of the experiment conducted in the US. The only differences with the experiment conducted in India were the stakes at play, which, in India, were exactly one third of those used in the US.

***Introductory screen***

Welcome to this HIT.

This HIT will take about 10-15 minutes. For the participation in this HIT, you will earn 90c. You will also earn additional money (a minimum of 36c).

This HIT consists of two parts.

In the first part, you will be asked to make several decisions. In this part, there is no correct or incorrect answer, you will be asked to choose the options you prefer. Your earnings from this part will depend on your decisions or the decisions of other participants.

In the second part, your earnings will depend only on your decisions.

IMPORTANT: at the end of the first part, we will ask four additional simple questions to make sure you understood the task. Each question has only one correct answer.  If you fail to correctly answer any of the four questions, the survey will automatically end and you will not receive any completion code and consequently you will not get any payment.

IF YOU SUBMIT THE TASK WITHOUT COMPLETION CODE, IT WILL BE REJECTED.

(here the subjects could either continue or end the survey)

***Social preferences elicitation (time pressure condition)***

*Screen 1.*

In the next screens, you will be asked to make six decisions.

You will have **only 5 seconds to make each choice**. A timer will appear at the bottom of the screen.

In each decision problem you will be paired with a different participant.

After the survey is completed, you and the other participants will be paid according to your choices. Specifically, each decision problem has two possible roles: one active and one passive. You will be paid for one single decision problem selected at random among the six; and within that decision problem your final role will also be randomly selected. No deception is used.

Each decision problem consists of choosing between two allocations of points between yourself and the other participant. Option A will be the same across the six decisions: 10 points for you and 10 points for the other participant. However, the allocation in Option B will change each time.

Each point will be converted into money according to the following exchange rate:

1 point = 9c, that is, 10 points = 90c

If you are ready, go to the next page.

*Screen 2.*

As mentioned, your earnings and the earnings of the person you have been paired with will depend on the option chosen by the one who is randomly selected as the allocator (active role) for the randomly selected decision problem.

If Option A was chosen in the selected decision problem, both individuals will receive 10 points. If Option B was chosen in the selected decision problem, your earnings and the earnings of the other person will be determined by the allocation specified in that decision problem.

Note that the other participant will never be informed of your personal identity and you will not be informed of the other participant’s personal identity.

Once you have finished reading these instructions, select "Continue" to see an example.

*Screen 3.*

Below there is an example. Note that in this example both options show the same allocation. In the real decisions, 'Option A' will always be the same, however, 'Option B' will change in each decision and will be different from the allocation in 'Option A'.

Please select one option, by clicking on the text describing that option. Once you have chosen one of the two options click on the button to go to the next screen.

Remember that you will have only 5 seconds to make each choice. A timer will appear at the bottom of the screen.

- Option A: 10 points for you and 10 points for the other participant
- Option B: 10 points for you and 10 points for the other participant

*Screen 4.*

It is now time to make your real choices.

Remember that you will have **only 5 seconds** to make each choice.

If you are ready, select "Continue' and go to the first decision problem.

*Screen 5.*

Select 'Option A' or ' Option B'

- Option A: 10 points for you and 10 points for the other participant
- Option B: 10 points for you and 6 points for the other participant

(here there was a clearly visible timer counting down from 5 to 0 – the same timer was present also in all subsequent decision screens. The survey did *not* automatically go to the next screen, when the timer reached 0. Thus participants were allowed to make their decision at any time)

*Screen 6.*

Remember that you will have **only 5 seconds** to make each choice.

Select "Continue' and go to the second decision problem.

*Screen 7.*

Select 'Option A' or ' Option B'

- Option A: 10 points for you and 10 points for the other participant
- Option B: 16 points for you and 4 points for the other participant

*Screen 8.*

Remember that you will have **only 5 seconds** to make each choice.

Select "Continue' and go to the third decision problem.

*Screen 9.*

Select 'Option A' or ' Option B'

- Option A: 10 points for you and 10 points for the other participant
- Option B: 10 points for you and 18 points for the other participant

*Screen 10.*

Remember that you will have **only 5 seconds** to make each choice.

Select "Continue' and go to the fourth decision problem.

*Screen 11.*

Select 'Option A' or ' Option B'

- Option A: 10 points for you and 10 points for the other participant
- Option B: 11 points for you and 19 points for the other participant

*Screen 12.*

Remember that you will have **only 5 seconds** to make each choice.

Select "Continue' and go to the fifth decision problem.

*Screen 13.*

Select 'Option A' or ' Option B'

- Option A: 10 points for you and 10 points for the other participant
- Option B: 12 points for you and 4 points for the other participant

*Screen 14.*

Remember that you will have **only 5 seconds** to make each choice.

Select "Continue' and go to the sixth decision problem.

*Screen 15.*

Select 'Option A' or ' Option B'

- Option A: 10 points for you and 10 points for the other participant
- Option B: 8 points for you and 16 points for the other participant

***Social preferences elicitation (baseline condition)***

Instructions were exactly the same as in the time pressure condition, apart from the fact that the sentences “You will have **only 5 seconds to make each choice**. A timer will appear at the bottom of the screen” and “Remember that you will have **only 5 seconds** to make each choice” were deleted and no timer appeared at the bottom of the decision screens.

***Social preferences elicitation (time delay condition)***

Instructions were exactly the same as in the time pressure condition, apart from the fact that the sentences “You will have **only 5 seconds to make each choice**. A timer will appear at the bottom of the screen” and “Remember that you will have **only 5 seconds** to make each choice” were replaced by “You will have to **wait for at least 15 seconds** before making each choice. Use this time to think carefully about the decision problem. A timer will appear at the bottom of the screen” and “Remember that you will have to **wait for at least 15 seconds**before making a choice.”, respectively.

***Comprehension questions (common to all conditions)***

(The following questions were presented in random order. For each question, there were two available answers: Option A and Option B. Subjects failing any of them were automatically redirected to the end of the survey and did not receive any completion code to claim for their payment.)

*Screen 1.*

Given the following decision problem:

- Option A: 10 points for you and 10 points for the other participant
- Option B: 16 points for you and 4 points for the other participant

Which is the choice by YOU that maximizes YOUR outcome?

*Screen 2.*

Given the following decision problem:

- Option A: 10 points for you and 10 points for the other participant
- Option B: 10 points for you and 18 points for the other participant

Which is the choice by YOU that maximizes the OTHER PARTICIPANT’s outcome?

*Screen 3.*

Given the following decision problem:

- Option A: 10 points for you and 10 points for the other participant
- Option B: 12 points for you and 4 points for the other participant

Which is the choice by YOU that maximizes the OTHER PARTICIPANT’s outcome?

*Screen 4.*

Given the following decision problem:

- Option A: 10 points for you and 10 points for the other participant
- Option B: 11 points for you and 19 points for the other participant

Which is the choice by YOU that maximizes YOUR outcome?

Numeracy Test

(All participants faced the following seven problems. As usual, the Numeracy score was defined as the number of correct answers).

Problem 1. Imagine that we filp a fair coin 1,000 times. What is your best guess about how many times the coin would come up tails in 1,000 flips?

Problem 2. A lottery ticket offers a 1% probability of winning a $10 prize. Imagine that 1,000 pople buy a ticket each. What is your best guess about how many people would win the $10 prize?

Problem 3. In a TV show, the probability of winning a car is 1 in 1,000. What percent of contestants in the TV show win a car?

Problem 4. Out of 1,000 students in a small university, 500 are in the business school. Out of these 500 students in the business school, 100 are male students. Out of the 500 students that are not in the business school, 300 are male students. What is the probability that a randomly drawn male student is in the business school? Please indicate the probability in percent.

Problem 5a. (shown only to subjects who solved Problem 4 incorrectly). Imagine we roll a fair five-sided die 50 times. On average, out of these 50 rolls, how many times would this fair five-sided dies show an odd number (1, 3, or 5)?

Problem 5b. (shown only to subjects who solved Problem 4 correctly). Imagine we are rolling a loaded die (6 sides). The chance that the dies shows a 1 is twice as higher as the chance of each of the other numbers. On average, out of 70 throws, how many times would the die show the number 1?

Cognitive Reflection Test

(All participants faced the following seven problems. As usual, the CRT score was the number of correct answers).

Problem 1. A table and a chair cost $150 in total. The table costs 100 dollars more than the chair. How much does the chair cost?

Problem 2. If it takes 10 mechanics 10 hours to fix 10 cars, how long does it take 80 mechanics to fix 80 cars?

Problem 3. A new library is purchasing books for its collection. Every week, the number of books acquired doubles. If it takes 36 weeks to buy all the books they need, how long does it take for the library to buy half of the books they need?

Problem 4. In the zoo, the lions eat one ton of meat every 6 weeks, and the tigers eat another ton of meat every 12 weeks. How long would it take them (lions and tigers) to eat one ton of meat together?

Problem 5. John obtained the 25^th^ fastest mark and the 25^th^ slowest mark in a race. How many people participated in the race?

Problem 6. An art collector acquires a famous painting for 50 million and sells it for 60 million. Some years later, the collector buys it back for 70 million, and sells it finally for 80 million. How much has the collector won in total?

Problem 7. Mary invested $12,000 in the stock market in November 2013. Six months later, on May 2014, the stocks she had purchased were down 50%. Fortunately for Mary, from May 2014 to August 2014, the stocks she had purchased went up 75%. At this point, Mary has

- 1. won money
  2. lost money
  3. neither won nor lost money
